# An Indication of Reliability of the Two-Level Approach of the AWIN Welfare Assessment Protocol for Horses

**DOI:** 10.3390/ani8010007

**Published:** 2018-01-05

**Authors:** Irena Czycholl, Kathrin Büttner, Philipp Klingbeil, Joachim Krieter

**Affiliations:** Institute of Animal Breeding and Husbandry, Christian-Albrechts-University Kiel, Olshausenstraße 40, 24118 Kiel, Germany; kbuettner@tierzucht.uni-kiel.de (K.B.); pklingbeil@tierzucht.uni-kiel.de (P.K.); jkrieter@tierzucht.uni-kiel.de (J.K.)

**Keywords:** animal-based, animal welfare assessment, feasibility, AWIN protocol, horses, reliability

## Abstract

**Simple Summary:**

Animal welfare is a very emotional issue. It is therefore necessary to measure it objectively. As welfare includes different components such as the health status, the behaviour and the emotional state, different indicators are needed for its assessment. A two-level approach is proposed in the Animal Welfare Indicators (AWIN) assessment protocol for horses; the first level providing a fast overview and the second more details. The aim of this study was to give an indication whether this two-level approach produces reliable results, i.e., whether the first level assessment does indeed provide a good overview or whether too many welfare issues remain undetected. Therefore, a trained observer performed 112 first and second level assessments directly following each other. The results were compared based on the agreement between the two levels. In this study, based on one observer, overall, the first level did provide a good overview of the welfare status. Adaption of some of the indicators of the first level assessment might be necessary. Nevertheless, this two-level approach enhances feasibility and there is indication that it is a reliable approach. Therewith, this approach might also be interesting for implementation in other welfare assessment schemes.

**Abstract:**

To enhance feasibility, the Animal Welfare Indicators (AWIN) assessment protocol for horses consists of two levels: the first is a visual inspection of a sample of horses performed from a distance, the second a close-up inspection of all horses. The aim was to analyse whether information would be lost if only the first level were performed. In this study, 112 first and 112 second level assessments carried out on a subsequent day by one observer were compared by calculating the Spearman’s Rank Correlation Coefficient (RS), Intraclass Correlation Coefficients (ICC), Smallest Detectable Changes (SDC) and Limits of Agreements (LoA). Most indicators demonstrated sufficient reliability between the two levels. Exceptions were the Horse Grimace Scale, the Avoidance Distance Test and the Voluntary Human Approach Test (e.g., Voluntary Human Approach Test: RS: 0.38, ICC: 0.38, SDC: 0.21, LoA: −0.25–0.17), which could, however, be also interpreted as a lack of test-retest reliability. Further disagreement was found for the indicator consistency of manure (RS: 0.31, ICC: 0.38, SDC: 0.36, LoA: −0.38–0.36). For these indicators, an adaptation of the first level would be beneficial. Overall, in this study, the division into two levels was reliable and might therewith have the potential to enhance feasibility in other welfare assessment schemes.

## 1. Introduction

Animal welfare has become a matter of a very emotional public and political debate in Western society [1,2]. Therefore, it has become necessary to find a way of measuring it on a scientific basis in order to objectify the discussion and guide political decisions [3]. The Welfare Quality^®^ protocols were developed and published in 2009 with exactly this aim in mind [4,5,6]. This project succeeded in developing a broadly accepted comprehensive definition of animal welfare. Furthermore, the focus was on animal-based measures [7], as only animal-based indicators can detect the true welfare status while management- or resource-based indicators remain a risk assessment of the husbandry conditions under which the animal lives [8]. These protocols provided welfare assessments for cattle, pigs and poultry. In the Animal Welfare Indicators (AWIN) follow-up project, based on the concept of Welfare Quality^®^, welfare assessment protocols were developed for turkey, sheep, goats, donkeys and horses [9,10,11,12,13]. In the Welfare Quality^®^ as well as the AWIN protocols, welfare is defined as a multidimensional complex made up of good feeding, good housing, good health and appropriate behaviour. Each of these dimensions has to be regarded separately, as compensation is not allowed [14]. Due to this multidimensionality, a combination of different indicators for the assessment of welfare is needed in order to take into account all the different aspects. 

However, assessing a lot of different indicators is time consuming. This is especially true as the assessment of animal-based indicators, on which one should rely in the terms of animal welfare, is usually more time-consuming than using resource- or management-based indicators. Thus, not surprisingly, studies concerning the Welfare Quality^®^ protocols especially have criticised a lack of feasibility, i.e., that an assessment takes too long [7]. For example, protocol assessments on growing pig farms usually take on average around five hours [15]. Although the general aim of the Welfare Quality^®^ project, i.e., that a protocol assessment should be completed within one day, has been reached [16], this time requirement hinders practical implementation with regard to, e.g., certification purposes or regular self-assessments by the farmers as the question of how to pay for that additional work load remains. 

In the AWIN follow-up project, the direct aim was to address the criticised issue of feasibility in order to simplify practical implementation. Therefore, keeping the multidimensional definition and still concentrating on animal-based indicators, a two-level approach was introduced into the AWIN welfare assessment protocol for horses in order to enhance its feasibility. Thereby, the first level was developed in order to provide a fast overview of the farm under assessment. Only if anything conspicuous is detected should a more detailed and also more time-consuming second level assessment be performed. The main difference between the two levels is that in the first level, only a sample of horses is assessed and the assessor only watches from the distance, while in the second level protocol the assessor touches and even interacts with the horse and each horse on the farm is assessed [11].

Despite this approach being very feasible, the question arose as to whether too much information would be lost if only the overview stage, i.e., the first level assessment, were conducted, thus whether welfare issues could really be safely detected. Specific testing for reliability has not hitherto been performed for all of the included indicators. The reliability of the first level assessment as such has not been evaluated at all. Thus, it is unknown whether it does indeed provide a good, reliable overview of the welfare status or else whether relevant information is lost if the more detailed second level stage is not applied. 

Nevertheless, reliability is one of the basic requirements for indicators as they need to be trustworthy and perform consistently well [17]. With on-farm assessment tools, the specific requirements usually include interobserver reliability, i.e., independence of the observer, and test-retest reliability, i.e., a certain consistency over time. However, in the assessment of the reliability of the two-level approach, the main interest is whether the first level as an overview stage actually adequately reflects the state of animal welfare detected by the more detailed second level. Thus, the results between the two protocols should point in the same direction and be in accordance with each other. This is why, in the present study, the agreement between the results of the first and the second level assessment of the AWIN protocol for horses was analysed in depth. In particular, the question was addressed as to whether the information gained from the first level assessment was in accordance with that of the more detailed second level. This reliability assessment is also of special importance for the Welfare Quality^®^ protocols whose feasibility could be greatly enhanced if this division into first and second level assessments proves to be reliable on-farm.

## 2. Materials and Methods

Data collection was carried out from November 2016 until August 2017. Fourteen farms in Northern Germany took part in the study on a voluntary basis. The farms were contacted either by their breeding associations or via phone calls. Farms were visited repeatedly by an assessor who was trained in the assessment in a three-day course by the developers of the AWIN protocol for horses. Each farm was visited eight times, whereby the visits on each farm were equally spread over the whole time period, i.e., three visits were carried out in winter, two in spring and three in summer on each farm. Each time, the first level assessment was carried out and on the following day, independently of the exact outcomes, the second level protocol. This procedure of repeated farm visits on the 14 farms resulted in a total of 112 first level (2160 assessed horses) and 112 second level (3448 assessed horses) protocol assessments, which were then compared. 

The farms were chosen to be quite different in order to enhance the variance of the study. Thus, the size ranged from 14 up to 120 horses. Four farms were mainly breeding stables, three were mainly sport stables (dressage and show jumping) and seven were mainly pension stables, i.e., mostly leisure horses were present. The breeds varied, but typically for Germany, German Warmbloods such as Holsteiner, Hanoverian and Trakehner dominated and made up about two thirds of the sample. The exact husbandry conditions varied also, ranging from pure single-box stabling to single paddock boxes to free stalls and group-housing. All horses were kept according to the national guidelines for horse-husbandry in Germany [18].

### 2.1. Protocol Assessments

A short overview of the first and second level assessments carried out in this study is presented in the following, a detailed overview of the indicators and their classification into the different categories can be found in the AWIN welfare assessment protocol for horses [11]. All assessments were carried out exactly following the instructions of the protocol and the training.

#### 2.1.1. First Level (Sample)

Each protocol assessment was carried out on a sample of horses on the farm to give a representative overview. The exact sample size for the first level assessment generally depends on the total number of horses present on-farm and is specified in the AWIN protocol for horses [11]. Here, the sample of horses was selected before entering the stable by means of an overview plan of the stable in which the boxes or places to be observed were marked. Thereby, where possible, assessment of the horses in boxes next to each other was avoided to counter influences on the reactions especially in the behavioural tests. It was not necessary to touch the horse during the whole procedure. However, if the horses in this study were wearing blankets, these were taken off before the start of assessment and put back on again afterwards.

The first level protocol assessment started with the evaluation of signs of pain by the application of the Horse Grimace Scale. The Horse Grimace Scale is a standardised method for the evaluation of pain by changes in the horses’ facial expression [19]. According to the protocol, the assessor determined in an undisturbed horse whether the ears were stiff and turned backwards, whether tension above the eye area or orbital tightening was present, whether the chewing muscles were strained and prominent, whether the mouth was strained and the chin pronounced and whether the nostrils were strained and the profile flattened. The assessor noted on a three-point scale for each of these six regions whether these signs were not present at all, moderately present or obviously present. If the horse was for example eating or sleeping, it might not have been possible to carry out the Horse Grimace Scale correctly. In such cases, a non-assessable score was given. 

The horse was then observed and any signs of stereotypies noted, i.e., repetitive, relatively invariant behaviour with no obvious function (especially crib-biting, weaving, head nodding, wood chewing). This was scored on a two-point scale as absent or present. 

Afterwards, an Avoidance Distance Test was performed by the assessor approaching the horse in a standardised way from outside the box if stabled, and assessing whether avoidance behaviour was shown or not at any stage. After this, a hand was presented to the horse and the assessment scored whether the horse approached the hand voluntarily (Voluntary Human Approach Test). The Voluntary Human Approach Test assesses whether negative signs, no interest or positive signs are shown. 

Still from outside the box, i.e., a distance of approximately 2–3 m, by just watching the animal from the front to the back, the Body Condition Score was then evaluated by assessing the fat and muscle covering of prominent bones, thereby applying a five-point scale with 1 being too thin, 3 the optimum and 5 being too fat. Furthermore, any abnormalities in the hair coat condition, abnormal breathing, swollen joints, ocular, nasal or genital discharges and signs of uterine prolapse were assessed on a two-point scale (absence or presence). For the assessment of integument alterations, the muzzle, head (including ears), neck (excluding withers), shoulder (including withers, excluding elbow), midsection (back, loin, flank, barrel), legs (including elbow, stifle, pastern, excluding coronet) and hooves (including coronet) were all assessed separately for lesions >2 cm² or lesions >4 cm length. Lesions were thereby separated into alopecia, a superficial skin lesion, deep wound or swelling. 

Afterwards, the box was entered to look for signs of hoof neglect, i.e., overgrown, rarely trimmed or incorrectly trimmed hooves and to assess the consistency of manure present each on a two-point scale. Some resource- and management-based indicators were also noted. However, since these were not taken into account in the present study, these are not described further here.

#### 2.1.2. Second Level (All)

The second level protocol of this two-level approach describes the assessment of each animal on the farm. The second level protocol also starts from outside the box with the assessment of the Horse Grimace Scale and stereotypies. Therefore, these indicators were assessed in this study in the same way as in the first level protocol. The first part of the Qualitative Behaviour Assessment was carried out as an additional indicator, at this stage for 30 s. This is a free behavioural observation, i.e., the observer simply watches the horse for the given time. After the second part, which was performed after entering the box later on, the impressions of the observer were noted for a given list of adjectives as follows: aggressive, alarmed, annoyed, apathetic, at ease, curious, friendly, fearful, happy, looking for contact, relaxed, pushy and uneasy. For each of these adjectives, a 125 mm Visual Analogue Scale was used for each individually assessed horse to note whether the term was found to be absent (0 mm) or dominant (125 mm). Afterwards, the Avoidance Distance Test and the Voluntary Animal Approach Test were carried out, also as described in the first level protocol. Furthermore, it was noted whether coughing occurred during a period of 5 min.

In contrast to the first level, the box was already entered in the following. First, two additional indicators, namely a Forced Human Approach Test and another Qualitative Behaviour Assessment were performed. For the Forced Human Approach Test, the horse was approached slowly and, if possible, the left side of the horse was touched from the neck over the back down towards the tail. It was also assessed whether at any stage negative signs, avoidance or positive signs were demonstrated. For the second part of the Qualitative Behaviour assessment, the horse was scratched at the whither for 30 s as an imitation of allo-grooming behaviour. The expressive behaviour of the horse during this manipulation was also assessed. Both parts of the Qualitative Behaviour Assessment were taken into account when filling out the Visual Analogue Scales for the given adjectives. Following this, the Body Condition Score, hair coat condition, abnormal breathing, nasal, ocular and genital discharges, uterine prolapse and consistency of manure were assessed from inside the box. The main difference to the first level assessment was that the animals could be touched, e.g., for the assessment of the Body Condition Score.

Afterwards, the horse was led outside the box by a handler for a lameness inspection, which is assessed on a three-point scale (unable to stand up, lame, not lame). Being held on a halter allowed the indicators integument alterations, swollen joints and signs of hoof neglect to be assessed by a close-up inspection. Lesions at the mouth corners were also assessed as additional indicators on a two-point scale (absent or present). 

The horse was then brought back into its box, where a fear test was performed using a green 1.5 L plastic bottle filled with small stones and a 4 m string attached to it. The bottle was then hung in the box. The time the horse took to approach the bottle was measured as the first latency time. When it touched the bottle or after a total time of 300 s, the bottle was dropped to the floor and the time the horse took to re-approach the bottle was measured as the second latency time. The test was terminated if the horse did not re-approach within another 300 s. As in the first level assessment, some resource- and management-based indicators were additionally assessed which, however, are not further described here as they were not included in the analysis.

### 2.2. Statistics

The AWIN welfare assessment protocol for horses aims to evaluate the welfare status of a farm. To do so, the results are expressed at farm level as percentages of animals sorted into the dedicated categories of the respective indicator. Hence, in this study, all calculations were performed with continuous data (percentages of animals). Only the animal-based indicators and only those assessed in the first as well as in the second levels were compared. These indicators are demonstrated in Table 1.

In these comparisons, the different categories of each single indicator were treated as independent variables. Thus, for example, six sub-indicators were assessed in the analysis of the indicator discharges; these were ocular discharge (0), ocular discharge (1), nasal discharge (0), nasal discharge (1), genital discharge (0), genital discharge (1). If any indicator was scored as non-assessable in either of the levels of the protocol, e.g., if a horse was sleeping and thus an assessment of the Horse Grimace Scale on that day was not possible, these cases were excluded from the comparisons.

#### Statistical Parameters

For the comparisons, a combination of different reliability and agreement parameters was calculated with the statistical software SAS^®^ 9.4 (SAS Institute Inc., Cary, NC, USA) [20]: Spearman’s Rank Correlation Coefficient (RS), Intraclass Correlation Coefficient (ICC), Smallest Detectable Change (SDC) and Limits of Agreement (LoA). This combination of parameters was chosen as the data were not normally distributed. However, variance homogeneity was present which was tested with a Levene test beforehand. The applied formulas for the statistical parameters are explained in the following. 

The RS is a non-parametric measure of rank correlation and is quite commonly used in animal welfare science [21,22]. The values can differ between −1 and 1. Correlation is better the closer the value is to 1. Negative values point out negative correlations. In accordance with the suggestions of Martin and Bateson [23], an RS equal to or greater than 0.4 was interpreted as acceptable correlation and RS equal to or greater than 0.7 as good correlation.

The ICC is based on an analysis of variance carried out by the following one-way model in accordance with the suggestions of Shrout and Fleiss [24]:

(1) $$X_{\mathrm{jk}}=\varepsilon +\alpha_{j}+\varepsilon_{\mathrm{jk}}$$

with X_jk_ being the measured value, µ the general average value for each assessed indicator category, α_j_ the random effect of the difference between the study objects (112 farm visits) and ε_jk_ the general error term. The ICC was then calculated by putting into proportion the variance of the same subject (levels of the protocol) to the total variance [25] by the following formula in accordance with de Vet et al. [26]:

(2) $$\mathrm{ICC}=\frac{\sigma_{\left(\mathrm{objects}\right)}^{2}}{\sigma_{\left(\mathrm{objects}\right)}^{2}\;+\;\sigma_{\left(\mathrm{residual}\right)}^{2}\;}$$

with *σ*² representing the variance of the study objects and the residual variance, respectively. Corresponding to this formula, the ICC can reach values between 0 and 1 [27]. As proposed by McGraw and Wong [28], an ICC equal to or greater than 0.4 was interpreted as acceptable reliability and an ICC equal to or greater than 0.7 as good reliability.

The SDC is an expression of the measurement error, which is derived from the above-named formulas. For the SDC, the measurement error contains the variance of the levels of the protocol and the residual variance. According to de Vet et al. [26], the SDC is calculated by

(3) $$\mathrm{SDC}=1.96*\sqrt{2}*(\sigma_{\left(\mathrm{observer}\right)}^{2}+\sigma_{\left(\mathrm{residual}\right)}^{2})$$

The SDC outputs the smallest change in the score that can be detected despite the measurement error [29]. The values of the SDC are the same as the measurement unit of the assessed indicators, i.e., percentages in this study. Based on the interpretation of the simple agreement coefficient in de Vet et al. [26], a SDC smaller than or equal to 0.1 distributing a deviation of up to 10% was interpreted as acceptable agreement and values smaller than or equal to 0.05 distributing a deviation of up to 5% as good agreement.

The LoA were also calculated according to de Vet et al. [26] by the formula:

(4) $$\mathrm{LoA}=\mathrm{mean}\pm 1.96*\sqrt{2}*\sigma_{\left(\mathrm{residual}\right)}^{2}$$

The LoA, which was first introduced by Bland and Altman [30], calculates the range of the difference between two sets of measurement values (first and second level protocols). In this study, it is expressed as relative frequency, thus ranging from −1 to 1. The direction of −1 is due to deviations in the first level assessment, the direction of 1 to deviations in the second level assessment. Interpretation was again based on the simple agreement coefficient of de Vet et al. [26] and therefore an interval smaller than or equal to −0.1 to 0.1 was interpreted as acceptable and −0.05 to 0.05 as good agreement.

For interpretation, again according to the suggestions of de Vet et al. [26], it was determined that all parameters had to reach these predefined acceptability limits in order for the evaluation of that indicator in the first level protocol to be interpreted as reliable.

### 2.3. Ethical Statement

All horses were normally farmed animals. The disturbance of the animals is, by nature of the AWIN protocol, kept to a minimum as it was invented to fit well into the normal farm routine. The authors declare that the experiments were carried out strictly following international animal welfare guidelines thereby adhering to the “German Animal Welfare Act” (German designation: TierSchG), the “German Order for the Protection of Animals used for Experimental Purposes and other Scientific Purposes” (German designation: TierSchVersV) and the “German Order for the Protection of Production Animals used for Farming Purposes and other Animals kept for the Production of Animal Products” (German designation: TierSchNutztV) were applied. No pain, suffering or injury was inflicted on the animals during the experiment.

## 3. Results

The time needed for a first level protocol assessment was on average six minutes plus or minus two per horse. The total time per farm ranged between 70 and 205 min with an average of 90 ± 35 min in this study. For the second level protocol, the time per horse varied between 17 ± 6 min. The total time per farm varied between 130 min and 660 min with an average of 245 ± 131 min in this study.

The mean values of the percentages of affected animals for those animal-based indicators that are used in the first as well as in the second level are presented in Table 2. These are presented as means of means, i.e., first, the means were calculated for the separate farm visits and from these means, the means shown in the table were calculated. This was done in order to present a short overview of the prevalence of each single indicator. Furthermore, in Table 2, the reliability and agreement parameters calculated for the comparison of the results of the two protocols are shown. Some of the indicators did not appear at all or the prevalence was so low that a calculation of the statistical parameters was not possible. These are left blank. Overall, one can summarise from Table 2 that the threshold values were met by the statistical parameters for most of the indicators. 

An exception has to be made for the Horse Grimace Scale. As can be seen from Table 1, for none of the different categories of the indicator did all four statistical parameters meet the threshold values. The SDC and LoA met the thresholds only for the scoring of the strained, prominent chewing muscles, strained mouth and pronounced chin as obviously present. RS and ICC were above the threshold value of 0.4 for the absence of orbital tightening. Only the RS was above the threshold value for the scoring of the strained, pronounced chewing muscles as absent or moderately present. 

Similarly, none of the four statistical parameters met the defined threshold values for the consistency of manure indicators.

For the Avoidance Distance Test, the parameters RS and ICC were above the value of 0.4 for the evaluation as non-avoidance, i.e., absence, but the parameters SDC and LoA did not meet their thresholds. Basically, the same is valid for the Voluntary Human Approach Test, for which only the two parameters RS and ICC were above 0.4 for the categorisation of positive signs. For the Body Condition Score, the limits of acceptability were also slightly exceeded for the SDC and LoA in the scoring as normal (category 3) and fat (category 4). The same is to be said for the indicators swollen joints, signs of hoof neglect as well as for the integument alterations alopecia on the shoulder and legs as well as swellings on the legs. 

Only SDC and LoA suggested agreement for nasal discharges, deep wounds on the head, swelling in the neck and skin lesions and deep wounds on the hooves, while RS and ICC were smaller than 0.4.

## 4. Discussion

### 4.1. Feasibility of the AWIN Welfare Assessment Protocol for Horses in General

It was possible to complete both levels within one day. The total time needed strongly depended on the total farm size for both levels. Furthermore, the time needed per horse rose for both levels if blankets had to be removed beforehand to assess the indicators on a particular horse and then put back on again afterwards. For the second level assessment, the time needed also varied due to different reactions in the fear test. Furthermore, the assessment took particularly long if the preparation for the handling of the horse was not well organised, e.g., if no halters to handle the horse were directly available or if the assessor had to wait for a person to handle the horse. Hence, good organisation and contact with the farmer beforehand was necessary to facilitate a fast and uncomplicated inspection of the farm.

Overall, the time needed for both levels of the AWIN welfare assessment protocol for horses can be interpreted as feasible. Even on large farms of more than 100 horses, the total time needed for the detailed second level protocol assessment did not exceed one day. According to Knierim and Winckler [16], the protocol can be interpreted therewith as feasible. Feasibility is greatly enhanced by the procedure of splitting the protocol into two levels, as the first level assessment significantly reduces the time needed per farm. 

The time needed per horse for the protocol assessments was within the bounds also stated in the AWIN welfare assessment protocol for horses [11] and are comparable to the times found by Forkman et al. [31] in a practical implication study on the AWIN welfare assessment protocol for horses carried out on farms in Germany and Italy.

### 4.2. Comparison between First and Second Level

Overall, the results demonstrated that there is quite good agreement between the first and the second level assessments. Exceptions to this were the Horse Grimace Scale, the Avoidance Distance Test, the Voluntary Human Approach Test and consistency of manure.

Two of the components of the Horse Grimace Scale (tension above the eye area as well as strained nostrils and flattened profile), uterine prolapse and deep wounds on the muzzle, shoulder and hooves occurred only very rarely or not at all in both protocol assessments. Thus, a comparison of the outcomes of the two levels of the protocol was not possible in this study. 

For the Horse Grimace Scale, sufficient agreement was detected consistently by the statistical parameters only for the scoring of the strained, pronounced chewing muscles as well as strained mouth and pronounced chin as being obviously present. All statistical parameters did not suggest agreement for any other components. Thus, overall, insufficient agreement was detected for the Horse Grimace Scale. Similar results were obtained for the comparison of the Avoidance Distance Test and the Voluntary Human Approach Test, for which agreement was not revealed consistently by the statistical parameters. However, regarding the execution of the assessment of the Horse Grimace Scale and the two behavioural tests, differences are rarely found between the procedure of assessment in the first and second level [11]. Thus, this disagreement rather hints at a low test-retest reliability in these tests than towards a bad agreement between the two protocols. Especially in terms of the two behavioural tests, this could be due to the fact that the animals remembered the test from the day before and thus were, e.g., rather uninterested or else less frightened on the second day as they remembered that basically nothing particularly positive or negative would happen [32,33]. This is even more valid since 112 individual farms were not visited only once in this study but were visited repeatedly. Most of the horses remained on the farms in between the data collection. These horses were thus assessed repeatedly at least for the second level assessment, in which all horses were assessed. This means the horses were tested eight or even more times (when they were also included in the first level assessment) with the same test. Changes in the reaction to the test might thus be well explained by potential learning or habituation effects. For example, [34] also describes habituation effects in sows that were retested in the same behavioural tests two weeks later. For the Horse Grimace Scale, it might be that too many disturbances due to feeding behaviour occurred which led to the differences in the assessment on the two follow-up assessments. Moreover, according to Thatcher et al. [35], pain faces are only valid for ≤48 h after the painful event and also probably not valid for the assessment of chronic pain. Furthermore, it has been reported that mild pain may be difficult to assess [19]. Thus, the question remains of whether this method is valid and reliable enough to be included in a general welfare assessment tool. This will be considered in a detailed test-retest reliability study in the future.

Good agreement was revealed by the statistical parameters for stereotypies, however, for which the assessment procedure was also quite alike in the two levels of the protocol. This can be interpreted as very good test-retest reliability for this indicator.

None of the statistical parameters suggested agreement for the indicator consistency of manure. This can most probably be explained by the differences in the precision of the assessment in the first and second levels. This means that for this indicator, information might be lost and therewith welfare issues overseen if only the first level is applied. The accuracy in this case probably also depends on the exact bedding material and thus, time should be taken to closely investigate whether manure is present and of which consistency it is.

The reliability parameters RS and ICC suggested reliability for the indicators swollen joints, hoof neglect and alopecia on the shoulder and legs as well as swelling on the legs, belonging to the general assessment of integument alterations, while the agreement parameters SDC and LoA did not. This might be due to the fact that these indicators could be scored reliably, but not in absolute agreement between the two levels of the protocol, i.e., this ranking of farms would stay the same. This is especially conceivable, since the predefined limits of acceptability for the SDC and LoA were only exceeded slightly in most cases. Also for the Body Condition Score, the limits of acceptability were slightly exceeded for the SDC and LoA in the scoring as normal (category 3) and fat (category 4). It is especially noteworthy that the reliability parameters detected very good agreement of >0.90. Again, this means that reliability is given, while absolute agreement is not. In this case, this can be well explained by the fact that most horses in this study were either category 3 or 4 and that the transition between these two categories was probably quite fluid. However, it also shows that touching the horse in the second level protocol helped the assessor to clear up uncertainties when finding it hard to decide between the two categories. Most horses were most probably in exactly the fluid transition between being normal or slightly too fat as leisure horses especially are usually quite well fed [35,36]. An adjustment of the first level protocol could further enhance the exact agreement and thus also the overall credibility of the overview stage for these indicators.

Only SDC and LoA suggested agreement for nasal discharges, alopecia and deep wounds on the head, swelling in the neck and skin lesions on the hooves, while RS and ICC were very low. It is striking that the prevalence for these indicators was quite low. Given the nature of reliability parameters, i.e., RS and ICC, they are strongly dependent on the variance of the sample under study [26,27]. In contrast, agreement parameters such as SDC and LoA are not dependent on the variance. Thus, for the animal-based indicators described above, reliability is underestimated, while agreement is good. Although more studies are needed in order to enhance the sample size for a final evaluation, based on this explanation these indicators can still be interpreted as good. 

All other indicators were of at least acceptable agreement. Hence, overall, from 76 categories of indicators that were compared, only 17 were not of sufficient reliability in the comparison between the two levels of the protocol. Therefore, overall, it can be concluded that the approach chosen in the AWIN welfare assessment protocol for horses to divide the welfare assessment into a fast overview, first level assessment and a more detailed and time-consuming, second level assessment can be a reliable approach to enhance feasibility in animal welfare assessment schemes. It should also be assessed whether the inadequacies revealed in the 17 categories of indicators are caused by the differences in the assessment of the two levels or by other causes such as low test-retest reliability or insufficient validity. It might be helpful to reconsider the exact categorisation criteria for some of the indicators such as the Body Condition Score, in order to further enhance the reliability of the first level assessment. The question of whether and how much relevant information is lost if the additional indicators of the second level assessment are not applied still remains however. This will be addressed in a further study.

### 4.3. Statistics

For statistical analysis, the single categories were treated as independent variables in order to enhance comparability to other studies, e.g., Czycholl et al. [37] and Temple et al. [38], who assessed the reliability of the indicators of the Welfare Quality^®^ animal welfare assessment protocol for growing pigs and chose this approach, too. Other approaches such as summing up the categories of the three-point scale to a single scale of presence, as for example carried out in Kirchner et al. [39] always go along with a loss of information. 

In interpreting the results, it must also be borne in mind that the total number of 112 protocol assessments of each level was achieved only on 14 farms. This was inevitable due to the fact that it was a practical on-farm study and the data was also to be used to analyse the test-retest reliability of the indicators of the AWIN welfare assessment protocol for horses. To balance this, the present farms were chosen to be of large variability. Each visit was treated as a separate assessment. However, the influence of seasonal effects can be neglected due to the study design: the first level assessment was carried out on one day and the second level assessment on the following day and these results were compared with each other.

Furthermore, one should consider that the study sample was limited to Northern Germany and only one observer. Further studies with more observers, e.g., carried out in other countries, are needed before the present findings can be generalised. An exact number of observers needed in order to be able to make a general assumption about reliability of the two-level approach can currently not be provided. This is due to the fact that the variance amongst observers using the AWIN protocol for horses still remains unknown. Studies using the completed AWIN protocol for horses are rare. There is, up to now, no knowledge about the interobserver reliability. Thus, before generalisation of the results, replication studies need to be carried out. Nevertheless, regarding the fact that this is the first study to address the question as to which degree the separation into two levels still produces trustworthy results, the present study provides a first indication of reliability of the two-level approach. Therewith, the study contributes to knowledge concerning welfare assessment tools and the insights especially contribute to the enhancement of feasibility. 

A range of different reliability and agreement parameters were calculated to answer the research question. This was done according to the suggestions of de Vet et al. [26], as each statistical parameter has its own advantages and disadvantages. For example, the RS and the ICC as reliability parameters always depend on the total variance of the study objects. Thus, if the variability becomes small, reliability will be underestimated. The SDC and LoA on the other hand are parameters of agreement and are not dependent on the variability of the study objects. However, these parameters assume agreement as exact agreement, while reliability also accepts those cases in which the exact scoring might differ, but the direction always stays the same [27] and, therewith, the ranking of the farms stays the same. Thus, reliability parameters assess how well study objects, i.e., in this case the farms or rather farm visits, can be distinguished from each other, despite measurement errors. Agreement parameters concern the measurement error and assess how close the scores for repeated measurements are. For example, if the assessment were always a bit stricter by the use of the second level protocol but were more consistent, this would still be reliable while not being an exact agreement between the two levels of the protocol. However, the problem of the agreement parameters is that subjectivity in their interpretation always remains. Furthermore, this combination of parameters was also used in other reliability assessment studies concerning welfare assessment of animals and, thus, their use is helpful in terms of standardisation and comparability to literature [37,38]. 

## 5. Conclusions

The present study aimed at giving a first indication on whether the less detailed overview, first level version of the AWIN welfare assessment protocol for horses provides a sufficient overview of the welfare status compared to the more detailed but also more time-consuming, second level assessment based on the results of one observer. Before generalisation of these results, replication studies need to be carried out. In this study, for most of the indicators, it was revealed that there is sufficient agreement between the two versions of the protocol and thus that the first level assessment does provide a good overview of the welfare status. Exceptions have to be made for the Horse Grimace Scale, the Voluntary Human Approach Test and the Avoidance Distance Test. However, this rather hints at insufficient test-retest reliability due to the procedure of assessment of these indicators. Insufficient agreement was further detected for the indicator consistency of manure. Thus, for this indicator in particular, it was revealed that a more detailed assessment might be necessary to detect its true prevalence. Furthermore, for some indicators (swollen joints, hoof neglect and alopecia on the shoulder and legs as well as swelling on the legs) only sufficient reliability was found, but not exact agreement. Thus, after further analyses of these indicators regarding, e.g., their interobserver and test-retest reliability, an adaption of the first level protocol for these indicators might be advisable. This is important information regarding the enhancement of the AWIN welfare assessment protocol for horses in the future. Some indicators were not observed at all or only very rarely (Body Condition Score 1 and 5, uterine prolapse and deep wounds on the muzzle, shoulder and hooves) and some indicators were observed with a very low variability amongst the farms in this study (nasal discharges, alopecia and deep wounds on the head, swelling in the neck and skin lesions on the hooves). Thus, a revaluation after an expansion of the sample size is necessary for these indicators. However, overall, the first level assessment was in this study capable of providing a reliable overview of the welfare status, although some adjustment and revision will be necessary. Hence, in order to enhance feasibility also of the Welfare Quality^®^ animal welfare assessment protocols as well as other welfare assessment schemes, the adoption and adaptation of a two-level approach might be a chance to enhance feasibility and therewith improve the possibilities for their practical implementation.

## Figures and Tables

**Table 1:** Animal-based indicators and their respective categories of the Animal Welfare Indicators (AWIN) welfare assessment protocol for horses.

Indicator	Categories (Code)
Horse Grimace Scale	
Ears stiff, turned backwards	Absent (0), moderately present (1), obviously present (2)
Tension above the eye area	Absent (0), moderately present (1), obviously present (2)
Orbital tightening	Absent (0), moderately present (1), obviously present (2)
Strained, prominent chewing muscles	Absent (0), moderately present (1), obviously present (2)
Strained mouth, pronounced chin	Absent (0), moderately present (1), obviously present (2)
Strained nostrils, flattened profile	Absent (0), moderately present (1), obviously present (2)
Stereotypies	Absent (0), present (1)
Avoidance Distance Test	No avoidance (0), avoidance (1)
Voluntary Human Approach Test	No interest (0), negative signs (1), positive signs (2)
Body Condition Score	Too thin (1), thin (2), normal (3), fat (4), too fat (5)
Hair coat condition	Normal (0), abnormal (1)
Abnormal breathing	Absent (0), present (1)
Swollen joints	Absent (0), present (1)
Discharge	
Ocular	Absent (0), present (1)
Nasal	Absent (0), present (1)
Genital	Absent (0), present (1)
Uterine prolapse	Absent (0), present (1)
Integument alterations	
Muzzle	Alopecia (1), superficial (2), deep wound (3), swelling (4)
Head (incl. ears)	Alopecia (1), superficial (2), deep wound (3), swelling (4)
Neck (excl. withers)	Alopecia (1), superficial (2), deep wound (3), swelling (4)
Shoulder (incl. withers, excl. elbows)	Alopecia (1), superficial (2), deep wound (3), swelling (4)
Midsection (back, loin, flank, barrel)	Alopecia (1), superficial (2), deep wound (3), swelling (4)
Legs (incl. elbow, stifle, pastern, excl. coronet)	Alopecia (1), superficial (2), deep wound (3), swelling (4)
Hooves (incl. coronet)	Alopecia (1), superficial (2), deep wound (3), swelling (4)
Hoof neglect	Absent (0), present (1)
Consistency of manure	Normal (0), abnormal (1)

**Table 2:** Mean values (and standard deviations (SD)) of the percentages of affected animals for each animal-based indicator used in the first (level 1) and second level (level 2) as well as the calculated statistical parameters Spearman’s Rank Correlation Coefficient (RS) (and *p*-values), Intraclass Correlation Coefficient (ICC) (and confidence intervals (CI)), Smallest Detectable Change (SDC) and Limits of Agreement (LoA) for the comparison of the two levels. The typing style indicates poor (normal), acceptable (*italic*) and good agreement (**bold**).

Indicator ^1^	Mean (SD): Level 1	Mean (SD): Level 2	RS ^4^ (*p*-Value)	ICC ^4^ (CI)	SDC ^4^	LoA ^4^
HGS ^2^: ears 0	47.9 (±21.3)	45.0 (±21.1)	0.09 (0.63)	0 (0–0.48)	0.43	−0.38 to 0.44
HGS ^2^: ears 1	43.3 (±20.1)	49.8 (±20.7)	0.1 (0.08)	0.09 (0–0.47)	0.34	−0.41 to 0.28
HGS ^2^: ears 2	8.7 (±8.0)	5.1 (±4.9)	−0.18 (0.35)	0 (0–0.14)	0.23	−0.18 to 0.25
HGS ^2^: eyes 0	93.5 (±13.1)	95.2 (±13.3)	0.33 (0.08)	0.25 (0.22–0.44)	0.21	−0.21 to 0.17
HGS ^2^: eyes 1	6.4 (±4.0)	4.7 (±3.6)	0.33 (0.08)	0.25 (0.22–0.44)	0.21	−0.17 to 0.21
HGS ^2^: eyes 2	0 (±0)	0 (±0)				
HGS ^2^: orbita 0	55.8 (±22.7)	60.2 (±17.7)	*0.52 (0.004)*	*0.5 (0.19–0.60)*	0.32	−0.37 to 0.28
HGS ^2^: orbita 1	37.9 (±21.1)	35.0 (±16.1)	0.33 (0.07)	0.18 (0.12–0.56)	0.38	−0.35 to 0.4
HGS ^2^: orbita 2	6.2 (±6.1)	4.7 (±3.2)	0.35 (0.06)	0.39 (0.23–0.63)	0.16	−0.13 to 0.16
HGS ^2^: chewing muscles 0	63.6 (±20.9)	68.6 (±17.6)	*0.53 (0.03)*	0.16 (0.04–0.50)	0.41	−0.43 to 0.33
HGS ^2^: chewing muscles 1	34.5 (±20.2)	30.9 (±16.8)	*0.6 (0.0006)*	0.15 (0–0.35)	0.41	−0.34 to 0.41
HGS ^2^: chewing muscles 2	1.8 (±1.8)	0.4 (±0.3)	0.05 (0.81)	0.02 (0–0.55)	*0.06*	*−0.05 to 0.08*
HGS ^2^: mouth 0	64.5 (±24.4)	70.2 (±22.5)	0.07 (0.33)	0 (0–0.15)	0.48	−0.5 to 0.39
HGS ^2^: mouth 1	34.2 (±21.8)	28.6 (±19.9)	0.01 (0.97)	0 (0–0.15)	0.49	−0.4 to 0.52
HGS ^2^: mouth 2	1.2 (±1.1)	1.1 (±1.0)	0.1 (0.62)	0 (0–0.10)	*0.07*	*−0.07 to 0.08*
HGS ^2^: nostrils 0	78.4 (±20.1)	80.5 (±15.7)	0.32 (0.10)	0 (0–0.55)	0.52	−0.5 to 0.46
HGS ^2^: nostrils 1	21.5 (±17.7)	18.4 (±15.7)	0.32 (0.08)	0 (0–0.54)	0.5	−0.44 to 0.5
HGS ^2^: nostrils 2	0.1 (±0.1)	0.9 (±0.8)			0.03	*−0.06 to 0.04*
Stereotypies 0	98.3 (±2.5)	98.8 (±7.2)	0.72 (<0.0001)	0.7 (0–0.94)	0.03	−0.04 to 0.03
Stereotypies 1	1.6 (±1.5)	1.1 (±1.0)	0.72 (<0.0001)	0.7 (0–0.94)	0.03	−0.03 to 0.04
Avoidance Distance Test 0	91.9 (±6.9)	92.3 (±19.1)	*0.58 (0.04)*	*0.55 (0.11–0.76)*	0.18	−0.18 to 0.17
Avoidance Distance Test 1	8.1 (±6.9)	7.6 (±5.2)	0.28 (0.04)	0.2 (0.11–0.56)	*0.1*	−0.07 to 0.11
Voluntary Human Approach Test 0	20.4 (±17.2)	24.7 (±19.9)	0.38 (0.04)	0.38 (0.31–0.68)	0.21	−0.25 to 0.17
Voluntary Human Approach Test 1	4.9 (±3.5)	4.1 (±3.1)	*0.41 (0.02)*	0 (0–0.19)	0.16	−0.13 to 0.15
Voluntary Human Approach Test 2	74.5 (±10.0)	71.1 (±11.6)	*0.48 (0.008)*	*0.46 (0.16–0.59)*	0.2	−0.16 to 0.23
Body Condition Score 1	0.1 (±0.1)	0 (±0)			0	−0.01 to 0.01
Body Condition Score 2	1.3 (±1.2)	1.1 (±0.9)	*0.54 (0.002)*	*0.53 (0.39–0.73)*	0.04	−0.04 to 0.05
Body Condition Score 3	74.8 (±25.6)	77.9 (±24.5)	0.92 (<0.0001)	0.91 (0.73–0.99)	0.12	−0.15 to 0.09
Body Condition Score 4	23.2 (±15.4)	20.9 (±15.8)	0.91 (<0.0001)	0.93 (0.73–0.99)	0.11	−0.08 to 0.14
Body Condition Score 5	0 (±0)	0 (±0)				
Hair coat condition 0	1.5 (±1.2)	1.2 (±1.2)	0.76 (<0.0001)	*0.61 (0.40–0.81)*	0.05	−0.05 to 0.05
Hair coat condition 1	98.4 (±21.3)	98.8 (±27.8)	0.76 (<0.0001)	*0.61 (0.40–0.81)*	0.05	−0.05 to 0.05
Abnormal breathing 0	99.2 (±24.8)	99.3 (±27.1)	0.2 (0.29)	0.35 (0–0.40)	0.03	−0.04 to 0.03
Abnormal breathing 1	0.7 (±0.4)	0.6 (±0.5)	0.2 (0.29)	0.35 (0–0.40)	0.03	−0.03 to 0.04
Swollen joints 0	91.1 (±14.8)	93.8 (±24.3)	*0.57 (0.001)*	*0.54 (0.18–0.60)*	0.12	−0.15 to 0.1
Swollen joints 1	8.8 (±3.1)	6.1 (±3.3)	*0.57 (0.001)*	*0.54 (0.18–0.60)*	0.12	−0.1 to 0.15
Discharge: ocular 0	97.3 (±1.3)	96.9 (±3.0)	*0.61 (0.0004)*	*0.64 (0.26–0.70)*	*0.07*	*−0.06 to 0.07*
Discharge: ocular 1	2.6 (±1.3)	3.0 (±3.0)	*0.61 (0.0004)*	*0.64 (0.26–0.70)*	*0.07*	*−0.07 to 0.06*
Discharge: nasal 0	98.9 (±0.8)	98.9 (±1.1)	0.3 (0.11)	0.27 (0.14–0.35)	*0.06*	*−0.06 to 0.06*
Discharge: nasal 1	1.0 (±0.8)	1.1 (±1.1)	0.3 (0.11)	0.27 (0.14–0.35)	*0.06*	*−0.06 to 0.06*
Discharge: genital 0	99.8 (±0)	99.9 (±0.1)	*0.49 (0.02)*	*0.52 (0.32–0.86)*	0.01	−0.01 to 0.01
Discharge: genital 1	0.2 (±0)	0.1 (±0.1)	*0.69 (<0.0001)*	*0.52 (0.32–0.86)*	0.01	−0.01 to 0.01
Uterine prolapse 0	1 (±0)	1 (±0)				
Uterine prolapse 1	0 (±0)	0 (±0)				
IA ^3^: muzzle: alopecia	2.0 (±2.0)	3.0 (±2.7)	*0.64 (<0.0001)*	*0.66 (0.38–0.72)*	*0.06*	*−0.07 to 0.05*
IA ^3^: muzzle: skin lesion	0.5 (±0.1)	0.5 (±0.1)	*0.52 (<0.0001)*	*0.41 (0–0.62)*	0.03	−0.03 to 0.03
IA ^3^: muzzle: deep wound	0 (±0)	0 (±0)				
IA ^3^: muzzle: swelling	0.2 (±0.1)	0.4 (±0.2)	0.83 (0.008)	0.82 (0.62–0.84)	0.01	−0.01 to 0.01
IA ^3^: head: alopecia	14.6 (±10.2)	17.4 (±13.9)	*0.68 (<0.0001)*	*0.58 (0.55–0.67)*	0.17	−0.2 to 0.14
IA ^3^: head: skin lesion	2.3 ±2.0)	3.1 (±2.6)	*0.6 (<0.0001)*	0.7 (0.67–0.86)	0.05	*−0.06 to 0.04*
IA ^3^: head: deep wound	0.6 (±0.2)	0.4 (±0.1)	−0.06 (0.70)	0 (0–0.39)	0.02	−0.03 to 0.02
IA ^3^: head: swelling	1.5 (±1.2)	0.4 (±0.1)	*0.65 (0.0006)*	*0.41 (0.10–0.55)*	*0.1*	*−0.1 to 0.1*
IA ^3^: neck: alopecia	5.1 (±3.8)	4.8 (±2.9)	*0.69 (0.0002)*	*0.65 (0.57–0.91)*	*0.08*	*−0.08 to 0.08*
IA ^3^: neck: skin lesion	0.8 (±0.6)	0.1 (±0)	*0.47 (<0.0001)*	*0.48 (0.15–0.57)*	0.05	*−0.06 to 0.05*
IA ^3^: neck: deep wound	0.3 (±0.1)	0.1 (±0.1)	*0.67 (0.007)*	*0.41 0.10–0.68)*	0.02	−0.03 to 0.03
IA ^3^: neck: swelling	0.4 (±0.1)	0.1 (±0.1)	−0.06 (0.70)	0 (0–0.37)	0.03	−0.03 to 0.04
IA ^3^: shoulder: alopecia	5.3 (±4.9)	5.1 (±4.8)	*0.41 (0.01)*	*0.47 (0.25–0.64)*	0.11	−0.12 to 0.12
IA ^3^: shoulder: skin lesion	1.3 (±1.0)	1.3 (±0.8)	*0.56 (0.05)*	*0.45 (0.30–0.67)*	0.05	−0.05 to 0.05
IA ^3^: shoulder: deep wound	0 (±0)	0 (±0)				
IA ^3^: shoulder: swelling	0.2 (±0.1)	0.1 (±0.1)	*0.6 (0.001)*	0.81 (0.76–0.90)	0.01	−0.01 to 0.01
IA ^3^: midsection: alopecia	4.6 (±4.3)	4.0 (±3.6)	0.73 (0.001)	0.83 (0.79–0.91)	*0.07*	*−0.07 to 0.08*
IA ^3^: midsection: skin lesion	0.7 (±0.2)	0.9 (±0.3)	0.95 (0.05)	0.91 0.83–0.99)	0.01	−0.02 to 0.01
IA ^3^: midsection: deep wound	0.1 (±0.1)	0.1 (±0.1)	1 (0.001)	1 (0.88–1)	0	0 to 0
IA ^3^: midsection: swelling	0.1 (±0.1)	0.2 (±0.1)	0.72 (0.04)	0.83 (0.78–0.91)	0.01	−0.01 to 0.01
IA ^3^: legs: alopecia	9.2 (±8.7)	13.6 (±11.2)	*0.4 (<0.0001)*	*0.53 (0.41–0.75)*	0.15	−0.19 to 0.1
IA ^3^: legs: skin lesion	2.8 (±2.4)	2.9 (±2.2)	*0.45 (0.02)*	0.7 (0.44–0.75)	*0.06*	*−0.06 to 0.06*
IA ^3^: legs: deep wound	0.3 (±0.1)	0.3 (±0.1)	*0.63 (0.01)*	*0.64 (0.53–0.71)*	0.02	−0.02 to 0.02
IA ^3^: legs: swelling	8.9 (±8.3)	7.4 (±6.9)	0.81 (0.0003)	0.73 (0.66–0.83)	0.11	−0.1 to 0.13
IA ^3^: hooves: alopecia	0.6 (±0.4)	0.7 (±0.4)	*0.65 (0.001)*	*0.65 (0.59–0.75)*	0.02	−0.02 to 0.02
IA ^3^: hooves: skin lesion	0.1 (±0.1)	0.1 (±0.1)	−0.07 (0.15)	0 (0–0.44)	0.02	−0.02 to 0.02
IA ^3^: hooves: deep wound	0.1 (±0.1)	0 (±0)			0	−0.01 to 0.01
IA ^3^: hooves: swelling	0.1 (±0.1)	0.1 (±0.1)	1 (0.0001)	1 (0.76–1)	0	0 to 0
Hoof neglect 0	95.4 (±27.2)	96.2 (±25.5)	*0.4 (0.02)*	*0.45 (0–0.56)*	0.12	−0.15 to 0.13
Hoof neglect 1	4.5 (±2.2)	3.7 (±1.9)	*0.4 (0.02)*	*0.45 (0–0.56)*	0.12	−0.13 to 0.15
Consistency of manure 0	69.9 (±23.3)	69.7 (±26.0)	0.35 (0.06)	0.34 (0–0.38)	0.37	−0.37 to 0.39
Consistency of manure 1	29.9 (±23.3)	30.2 (±19.3)	0.31 (0.10)	0.38 (0.31–68)	0.36	−0.38 to 0.36

^1^ For definition of codes, see Table 1, ^2^ HGS = Horse Grimace Scale, ^3^ IA = integument alterations, ^4^ Thresholds considered acceptable: RS ≥ 0.4, ICC ≥ 0.4, SDC ≤ 0.1, LoA ϵ (−0.1;0.1).
